# A pilot-study to assess the feasibility and acceptability of an Internet-based cognitive-behavior group therapy using video conference for patients with coronary artery heart disease

**DOI:** 10.1371/journal.pone.0207931

**Published:** 2018-11-29

**Authors:** Tin-Kwang Lin, Pao-Ta Yu, Lian-Yu Lin, Ping-Yen Liu, Yi-Da Li, Chiu-Tien Hsu, Yih-Ru Cheng, Chun-Yin Yeh, Shu-Shu Wong, Shih-An Pai, Huey-Ling Shee, Chia-Ying Weng

**Affiliations:** 1 School of Medicine, Tzu Chi University, Hualien, Taiwan; 2 Division of Cardiology, Department of Internal Medicine, The Buddhist Dalin Tzu Chi General Hospital, Chiayi, Taiwan; 3 Department of Computer Science and Information Engineering, National Chung Cheng University, Chiayi, Taiwan; 4 Department of Internal Medicine, College of Medicine, National Taiwan University and National Taiwan University Hospital, Taipei, Taiwan; 5 Institute of Clinical Medicine, National Cheng Kung University, Tainan, Taiwan; 6 Division of Cardiology, Internal Medicine, National Cheng Kung University Hospital, Tainan, Taiwan; 7 Center of Clinical Psychology, The Buddhist Dalin Tzu Chi General Hospital, Chiayi, Taiwan; 8 Clinical Psychology Center, National Taiwan University Hospital, Taipei, Taiwan; 9 National Cheng Kung University Hospital, Tainan, Taiwan; 10 Department of Psychology, Wenzhou University, Wenzhou City, China; 11 Department of Psychology, National Chung Cheng University, Chiayi, Taiwan; 12 College of Law, National Chung Cheng University, Chiayi, Taiwan; Medical University of Vienna, AUSTRIA

## Abstract

**Background:**

Many patients with coronary artery heart disease are unable to access traditional psychosocial rehabilitation conducted face to face due to excessive travel distance. Therefore, this study developed and assessed the feasibility and acceptability of an 8-week Internet-based cognitive-behavior group therapy program, described the patterns of use and measured change in risk factors.

**Methods:**

This study adopted an online video conference system, JointNet, to maintain group interaction functions similar to face to face groups online, and also built an self-learning platform to deliver psychoeducation content and cognitive-behavior therapy related materials and homework. Forty-three out-patients were recruited in the pilot study, who then chose to participate in either the Internet-based cognitive-behavior group therapy or face to face group based on their preference. Fourteen patients were assigned to the waiting-list control.

**Results:**

Seventeen participants (17/43 = 39.5%) chose the Internet-based cognitive-behavior group therapy program. Among them, thirteen participants (13/17 = 76.5%) finished the program and were more male (92.3% vs. 50%), employed (53.8% vs. 35.3%), and had longer education duration (13.9 vs. 12.5 years) than the counterparts of the face to face group. Furthermore, they were highly motivated with average number of log-ins (66.5 time), website surfing time (950.94 min), reading frequency (78.15 time) and reading time (355.90 min) for the self-learning platform during eight weeks; and also highly satisfied (97%) with visiting the self-learning platform and video conferences. The treatment effectiveness of Internet-based cognitive-behavior group therapy was comparable with face to face one in reducing anxiety, hostility, respiration rate, and in improving vasodilation but not depression compared with the waiting-list control.

**Conclusion:**

These results indicated that the Internet-based group therapy program using video conference is feasible and acceptable for the psychosocial rehabilitation of patients with coronary artery heart disease, and provides an alternative for patients who are unable to obtain conventional psychosocial rehabilitation conducted face to face.

## Introduction

A growing number of epidemiological studies have supported the contribution of psychosocial risk factors to the incidence and prognosis of coronary artery heart disease (CAD) [1,2]. Hostility is associated with an increased number of CAD events in the healthy and patient populations [3]. Anxiety [4,5] and depression [6] are also associated with an increased CAD risk in both community and patient cohorts.

Whalley, Thompson, and Taylor’s [7]) comprehensive systematic review of randomized controlled trials (RCT) indicated that psychological interventions significantly reduced hostility, anxiety, depression, and cardiac death among patients with CAD. The efficacy of traditional cognitive-behavior group therapy (CBGT) conducted face to face (FTF) in reducing hostility and psychological distress is well established [8,9,10]. Gidron, Davidson, & Bata [11] conducted a CBGT program aimed at reducing hostility in patients with CAD; the results indicated that such treatment can effectively lower patients’ hostility and diastolic blood pressure in a resting state. Blumenthal, Sherwood, Babyak, Watkins, Waugh, Georgiades, et al. [12] included stress management in a CBGT program and demonstrated that patients with CAD experienced alleviated emotional distress and improved flow-mediated dilation function.

In Taiwan, Weng et al. [13] developed the CBGT protocol aimed at reducing hostility and anxiety for patients with CAD, which included eight 2-h sessions with the following components: (1) psychoeducation; (2) self-awareness; (3) biofeedback-assisted relaxation training; (4) cognitive therapy, including cognitive flexibility and positive thinking; (5) behavior therapy that fosters compassion toward oneself and others; and (6) social support. The results indicated that patients with CAD in the CBGT group experienced reduced anxiety, hostility, and respiration rate alongside improved vasodilation function indexed by blood volume amplitude (BVA) compared with the wait-list control (WLC) group.

Although CBGT programs conducted FTF have proved to be effective, many patients with CAD are unable to access them due to excessive travel distances, expenditures, and time costs. Therefore, new effective and efficient strategies must be developed to fulfill patients’ preventive care needs.

Review studies on the effectiveness of Internet-based interventions have indicated that users are satisfied and that they result in improved behaviors and clinical outcomes compared with nonusers for patients with CAD [14,15,16,17].

Tate and Zabinski [18] described two forms of Internet-based communication: asynchronous and synchronous. Asynchronous communication is when message transmission between people occurs nonsimultaneously; examples include texts, emails, website communities, and electronic bulletin boards. Synchronous communication is a more interactive form that is more similar to FTF communication; once members log in to a specific website at scheduled time, they are able to interact with each other, either by text messages or by Internet-based conference calls and video. The advantage of a synchronous communication program is that it provides immediate support and reinforcements from group members. Furthermore, it provides the features of a therapeutic group; members are able to clarify their distressed emotions, beliefs, and behaviors through interacting with psychotherapists and group members. This also sustains the manifestation of particular therapeutic techniques, such as cognitive construction, relaxation training, or roleplaying to achieve the treatment goals of CBGT.

The purpose of this study was to develop an Internet-based cognitive-behavior group therapy (iCBGT) program with both synchronous and asynchronous communication features. A pilot study was adopted to assess the feasibility and acceptability of it, and to explore the patterns of use and measure change in risk factors for patients with CAD.

## Methods

### Study participants

Outpatients with CAD were referred by cardiologists from cardiology clinics between August 2012 and July 2014. The inclusion criteria were patients 35–75 years old, with one of the following: (1) coronary artery disease noted by coronary angiogram or vascular computer tomography; (2) a history of percutaneous coronary intervention or coronary artery bypass surgery; (3) a history of myocardial infarction or unstable angina; (4) positive exercise electrocardiogram for myocardial ischemia; (5) positive stress radionuclide perfusion scan for myocardial ischemia or infarction; or (6) positive stress cardiac echography for myocardial ischemia.

Patients with an unstable coronary condition, New York Heart Association functional class III or IV congestive heart failure, arrhythmia, pacemaker rhythm, or other severe physical illness were excluded to ensure that all participants were in relatively stable physical condition and able to undergo the intervention programs. In addition, the group leaders were licensed clinical psychologists who performed the assessment to ensure that none of the participants had been diagnosed with schizophrenia, major depression, substance abuse, or dementia and were thus able to receive effective intervention with intact reality testing.

The Institutional Review Board of The Buddhist Dalin Tzu Chi (B10001005-2), National Cheng Kung University Hospital (A-ER-102-151), and National Taiwan University Hospital Research Ethics Committee (NTUH-REC No.: 201306066RINC) approved the study protocols, and written informed consent was obtained from each patient.

### iCBGT protocol

**Fig 1:**
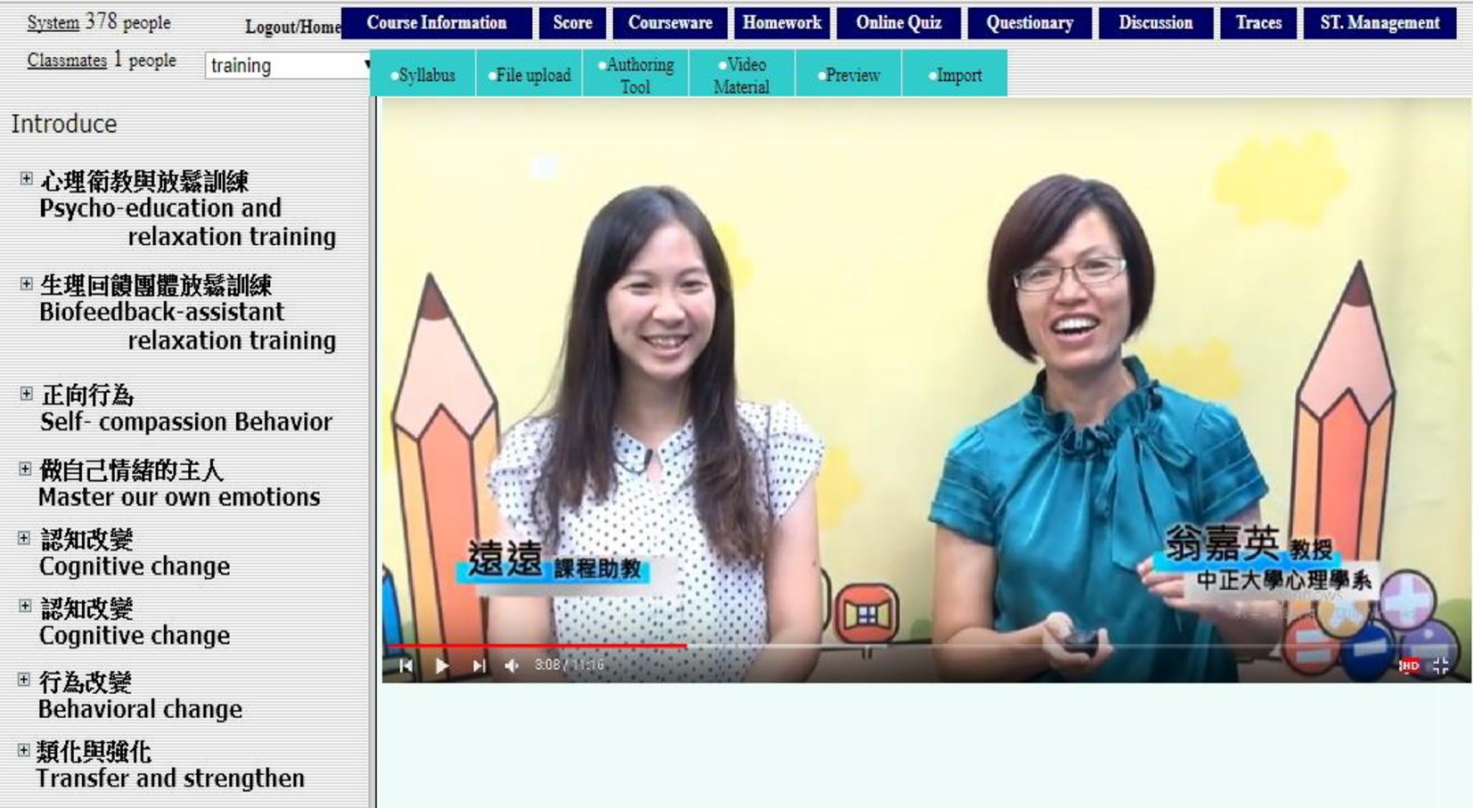
Self-learning platform with learning management system.

**Fig 2:**
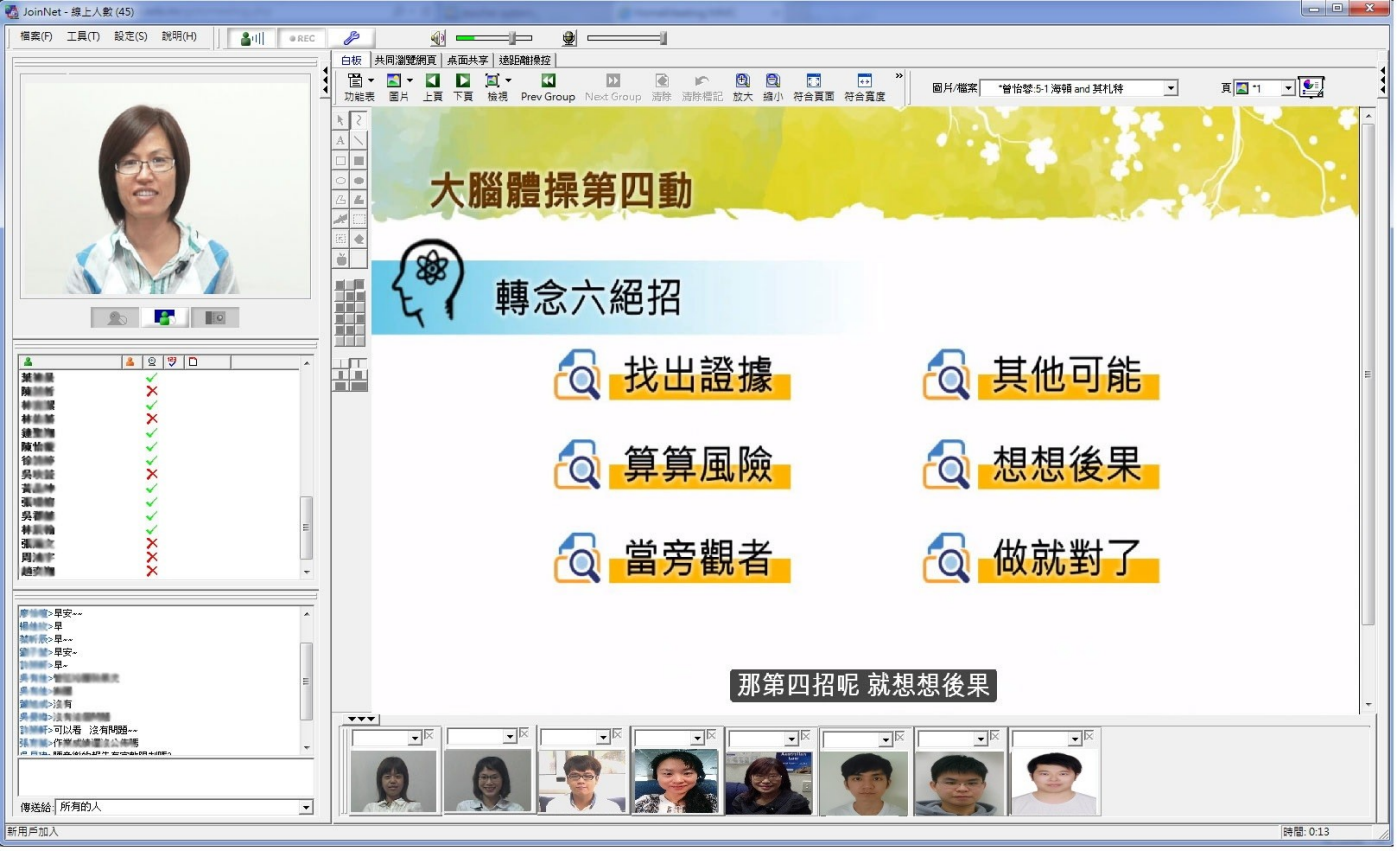
Online synchronous video conference with all the videos of participants.

The present study developed an iCBGT protocol modified from the FTF protocol [13] by adopting both asynchronous and synchronous communication forms. First, an asynchronous self-learning platform (SLP) was developed to deliver a psychoeducation curriculum that consisted of six 1-h streaming videos released over 8 weeks. The psychoeducational contents concerned the psychopathology of CAD, with main focus on how psychosocial distress affects the pathophysiological mechanism and prognosis of CAD through the autonomic nervous system, the hypothalamus-pituitary-adrenal axis, inflammation, platelet activation, and endothelial function. Along with Beck’s therapeutic interventions, self-monitoring, and homework assignments [19], these contents were delivered through text, presentations, pictures, clips, and animations. The asynchronous platform also sustained the interactions among participants and psychotherapists through emails, homework uploading and feedback, discussion forums, and electronic bulletin boards (Fig 1). Second, a synchronous online video conference system, JointNet version 5.9.2.1030 (HomeMeeting Inc., WA 98052, USA), was adopted to conduct the online group therapy using webcam videos for all the participants simultaneously (Fig 2). The online video conference (OVC) maintained the following key components [20] of group therapy: (1) group intimacy, by transmitting information about eye contact, smiling, and forward posture; and (2) interaction immediacy, by immediate verbal or nonverbal responses to shorten psychological distance among group members.

Table 1 lists both the FTF and the Internet-based CBGT protocol for each session. To increase group cohesion and sense of comradeship, the iCBGT program was started with two orientation sessions conducted FTF as in traditional group therapy. During these two sessions, training was provided to prepare participants for the level of technological readiness required to continue self-learning and participate in video conferences. To improve self-awareness and motivation to change, the results of psychological tests and psychophysiological stress profile (PSP) examined pre-intervention were interpreted by group leaders (clinical psychologists) in session 1. The PSP showed personal autonomic nervous system responses including increased heart rate and vasoconstriction during an anger recall task [21]. In addition, diaphragmatic breath relaxation facilitated by portable biofeedback equipment (StressEraser; Helicor, Inc., New York, USA), was provided for personal use in session 2.

**Table 1:** Comparison between the face to face and Internet-based cognitive-behavior group therapy protocol for each session.

		Face to Face	Internet-based		
			FTF	SLP	OVC
Session	Topic				
S1	Psychoeducation and relaxation training	1. Provide psychoeducation on the psychopathophysiology of CAD.	V	V	
		2. Provide and interpret the results of individualized PSP examined at preintervention.	V		
		3. Provide diaphragmatic breath relaxation training (slow, regular, stable, and smooth breathing).	V	V	
		4. Homework—practice diaphragmatic breath relaxation training.	V	V	
S2	Biofeedback-assistant relaxation	1. Provide psychoeducation on physical responses under stress.	V	V	
		2. Teach how to use the biofeedback equipment (StressEraser) for home diaphragmatic breath training.	V	V	
		3. Homework—practice diaphragmatic breath relaxation training.	V	V	
S3	Self- compassion behavior	1. Provide psychoeducation on the principle of behavioral competition.		V	V
		2. Teach group members to focus on the inside and develop new behaviors to care, treat, protect, and nourish themselves. Then, use the newly-learned self-compassion behaviors to substitute for harmful and unhealthy emotional compulsive behaviors.		V	V
		3. Homework—practice diaphragmatic breath relaxation training and self-compassion behavior.		V	V
S4	Master our own emotions	1. Provide psychoeducation on habitual emotional impulses of the associated neural circuits.		V	V
		2. Provide psychoeducation on the importance of self-monitoring of emotional reactions and enhancing strength of self-compassion behavior through repeat practice based on the principle of behavioral competition.		V	V
		3. Homework—practice diaphragmatic breath relaxation training & self-compassion behavior.		V	V
S5	Cognitive change	1. Provide psychoeducation on the impact of thoughts on emotional and behavioral responses.		V	V
		2. Teach CBT skills to increase cognitive flexibility and thought change.		V	V
		3. Homework—practice diaphragmatic breath relaxation training, self-compassion behavior, and thought change.		V	V
S6	Cognitive change	1. Experiencing change of emotional and behavioral reactions followed by change of thoughts through homework practice.			V
		2. Reinforce CBT skills to increase cognitive flexibility and thought changing.			V
		3. Describe the impact of cognitive and associated behavioral change on daily interpersonal situations.			V
		4. Facilitate the sharing of positive experiences to encourage the change of all group members.			V
		5. Homework—practice diaphragmatic breath relaxation training, self-compassion behavior, and thought change.		V	V
S7	Behavioral change	1. Review and reinforce behavioral change.			V
		2. Facilitate the sharing of effective strategies for behavioral changes in daily life.			V
		3. Provide psychoeducation on theory and strategies of emotion regulation.		V	V
		4. Facilitate the sharing of positive experiences to encourage the change of all group members.			V
		5. Appreciate group members for supporting each other to improve together.			V
		6. Homework—practice diaphragmatic breath relaxation training, self-compassion behavior, and thought and behavioral change.		V	V
S8	Transfer and strengthen	1. Review the effective CBT strategies for every participant in the group.	V		
		2. Facilitate the sharing of effective CBT strategies in daily life for the learning of all the group members.	V		
		3. Provide and interpret the results of individualized PSP with improved autonomic nervous system response examined postintervention to reinforce motivation to continue practicing.	V		
		4. Appreciate members and bless everyone to continue improving their physical and psychological health.	V		
		5. Homework—practice diaphragmatic breath relaxation training, self-compassion behavior, and thought and behavioral change	V		

CBT: cognitive-behavior therapy; FTF: face to face; OVC: online video conference; PSP: psychophysiological stress profile; SLP: self-learning platform

The following five sessions were conducted through the OVC. The participants of iCBGT were required to do the following tasks for five consecutive weeks from sessions 3 to 7:

1. Study the 1-h curriculum, do the homework assigned by psychotherapists in the digital curriculum, and then upload their homework practice experiences to the website prior to the OVC group session. The participants were permitted to study the curriculum anytime and anywhere with unlimited frequency and duration at their own pace.
2. Visit the OVC and interact with all the group members and psychotherapists together at the scheduled time.

Because the thought and behavior modification techniques were provided through both the SLP and book [22], with homework assigned every single session, the experiences of practicing these techniques were reinforced and coached in a timely fashion by the psychotherapists through the OVC group sessions. Furthermore, support from group members with the same afflictions was sustained by the OVC.

In the final session (session 8) conducted FTF, each participant was provided with his/her results of psychological tests and PSP examined at post-intervention, as well as a comparison of all the examinations between the pre- and post-intervention in session 8, to manifest personal progress and encourage them to continue practicing even after treatment cessation.

The iCBGT group leaders spent the same amount of time, that is, 2 h per week, performing 8-week group therapy, either through synchronous OVC in sessions 3 to 7 or through FTF in sessions 1, 2, and 8.

### Assessment procedure

All members in each group underwent the same assessment procedure before and after treatment to determine its effectiveness. Hostility, anxiety, and depression, as well as heart rate (HR), BVA, and respiration rate (RR) during the anger recall task were examined. After a 5-min seated rest period for adaptation, the participants went through the following four 5-min stages in the anger recall task: (1) baseline: sitting comfortably; (2) anger recall: recalling an anger event that happened in the past 6 months that still made them feel angry; (3) anger description: reporting the anger event; and (4) recovery: sitting comfortably and remaining silent.

Participants were instructed to take their medicine as usual, refrain from caffeinated beverages, alcohol, smoking, and excessive exercise 24 h prior to measurement and report to the laboratory at 9 AM. After finishing the psychological measurements, an anger recall task was administered to the participants in a sound-attenuated and temperature-controlled room.

#### Psychological measurements

Hostility was measured using the 20-item Chinese Hostility Inventory-Short Form [23], which contains four dimensions of hostility: (1) hostile cognition (α = .78); (2) hostile affection (α = .78); (3) expressive hostility (α = .76); and (4) suppressive hostility (α = .73). Cronbach’s α was .89 for the whole inventory. Anxiety was measured using the 20-item Chinese version of the trait anxiety subscale of the State-Trait Anxiety Inventory with α = .93 [24]. Depression was measured using the 21-item Chinese Version of the Beck Depression Inventory-II with α = .94 [25].

#### Psychophysiological measurements

Psychophysiological reactions including the HR, BVA, and RR of the participants during the anger recall task were collected using the BioGraph Infiniti version 5.0.3 (Thought Technology Ltd., Montreal, Quebec, Canada).

#### Internet usage behavior of participants

The asynchronous self-learning management system used in this study automatically recorded basic learner portfolios including the number of log-ins (login no.), the usage time of surfing on the website excluding reading time on the digital content (surfing time), the number of questions proposed on discussion forum (question no.), the frequency of reading the digital content (reading freq.), and the reading time on the digital content (reading time) over 8 weeks. JointNet recorded the whole live group processes. To better understand participants’ experiences on the synchronous conference environment, an evaluation form with four questions was designed to analyze learners’ satisfaction.

#### Statistical analysis

Group differences in demographic characteristics and research variables at study entry were compared using one-way analysis of variance (ANOVA) or chi-square test. For treatment effectiveness, two-way repeated-measures ANOVA with Bonferroni post hoc comparisons were used to examine the interaction effect of time*group on hostility, anxiety, depression, HR, BVA, and RR. Treatment effectiveness was calculated with partial eta squared (*η*_*p*_^*2*^). Small, medium, and large effects reflect in values of *η*_*p*_^*2*^ of 0.0099, 0.0588, and 0.1379, respectively [26]. All analyses were performed by using PASW Statistics for Windows, Version 18.0 (Chicago: SPSS Inc).

## Results

Forty-three patients without previous CBGT experience were recruited and then chose to participate in the iCBGT or FTF group based on their preference. Of these, 26 (60.5%) chose to participate in the FTF and 17 (39.5%) chose to participate in the iCBGT group. Eight participants (8/26 = 30.8%) in the FTF group dropped out for reasons of employment conflict or distance. Four participants (4/17 = 23.5%) in the iCBGT group dropped out, two because of the stress caused by using the Internet, one because a family member passed away, and one because of employment conflict. Finally, 18 patients (mean age = 62.72 ± 7.56, 50.0% male) completed the FTF program (at least 6 of 8 sessions), whereas 13 patients completed the iCBGT program (mean age = 58.23 ± 7.55, 92.3% male). Fourteen patients (mean age = 56.79 ± 9.76, 71.4% male) were assigned to the WLC group and received standard routine medical care without any clinical psychological treatment during their waiting-list period and then entered an 8-week CBGT program after their waiting-list period ended. There were no significant differences among the three groups at study entry in age, hostility, anxiety, depression, HR, BVA, and RR (Table 2). However, the male ratio of the Internet-based group was significantly higher than that of the FTF group.

**Table 2:** Demographic data, psychological variables and the physical activities at preintervention and postintervention.

	Whole sample (n = 45)			Internet-based group (n = 13)		Face to face group (n = 18)		Waiting-list control group (n = 14)		*F/χ*^*2*^	*F*	*η*_*p*_^*2*^
	*Mean*	*SD*		*Mean*	*SD*	*Mean*	*SD*	*Mean*	*SD*	pre-intervention	Group x Time	Group x Time
**Gender**	68.9% male			92.3% male		50.0% male		71.4% male		6.37*		
**Age**	59.58	8.54		58.23	7.55	62.72	7.56	56.79	9.76	2.25		
**Education years**	12.61	3.74		13.85	2.76	12.47	3.88	11.64	4.25	1.20		
**Employed rate**	50% employed			53.8% employed		35.3% employed		64.3% employed		2.69		
**Depression**												
*pre*	7.47	6.77		8.23	8.31	7.06	4.88	7.29	7.72	1.55	1.05	.05
*post*	5.87	6.79		4.85	5.89	5.44	5.32	7.36	9.14			
**Anxiety**												
*pre*	43.52	10.28		47.15	8.40	42.65	9.06	41.21	12.79	1.24	4.44*	.18
*post*	40.93	9.10		43.38	8.96	37.71	7.51	42.57	10.39			
**Hostility**												
*pre*	53.78	12.97		58.92	10.35	52.44	10.65	50.71	16.83	.12	2.98†	.12
*post*	50.04	12.38		54.15	9.21	46.56	10.40	50.71	16.26			
**Heart rate**^***a***^												
*pre*	70.65	8.86		68.83	8.11	73.64	10.16	67.93	6.24	1.30	.61	.03
*post*	69.52	11.03		67.74	12.65	71.13	10.31	68.97	10.82			
**BVA**^***a***^												
*pre*	5.63	3.89		4.53	3.09	5.20	4.02	8.08	4.00	2.43	3.21†	.15
*post*	7.44	4.99		8.37	5.93	7.20	4.84	6.60	4.05			
**Respiratory rate**^***b***^												
*pre*	14.73	1.97		14.26	2.14	14.91	2.33	14.94	1.24	.51	3.34*	.14
*post*	13.40	2.65		11.81	3.03	13.45	2.58	14.82	1.38			

BVA = Blood volume amplitude

a Average of four experimental stages

b Average of three experimental stages, the anger description stage was excluded due to speaking interference

† p < .10

* p < .05

### Satisfaction of Internet environment

The data for the asynchronous self-learning management system were in agreement with our expectation. The participants were highly motivated to visit the SLP, with a high average login no. (66.5 times), surfing time (950.94 min), reading freq. (78.15 times), and reading time (355.90 min) over 8 weeks. The only exception was the low question no. (1.77 times). Moreover, average satisfaction with the synchronous conference environment was more than 97%. Specifically, they were all impressed that they were able to remain at home or anywhere to receive treatment without worrying about the traffic problem or other issues that arise when traveling for treatment.

### Treatment effectiveness

Table 2 showed the results of psychological characteristics, there was a significance interaction effect of Time*Group in anxiety (*F*_*(2*, *41)*_ = 4.44, *p* < .05, with a large effect size [*η*_*p*_^*2*^ = .18]) and a marginal effect on hostility (*F*_*(2*, *42)*_ = 2.98, *p* = .06, with a medium effect size [*η*_*p*_*2* = .12]), but not on depression (*F*_*(2*, *42)*_ = 1.05, *p* = .36). The simple main effect showed a significant decrease on anxiety in the FTF group (*F*_*(1*, *41)*_ = 11.16, *p* < .001 with a large effect size [*η*_*p*_^*2*^ = .21]) and in the Internet-based group (*F*_*(1*, *41)*_ = 4.97, *p* < .05 with a medium effect size [*η*_*p*_^*2*^ = .11]), but not in the WLC group (*F*_*(1*, *41)*_ = .69, *p* = .41); it also showed a significant decrease on hostility in the FTF group (*F*_*(1*, *42)*_ = 12.70, *p* < .001 with a large effect size [*η*_*p*_^*2*^ = .23]) and in the Internet-based group (*F*_*(1*, *42)*_ = 6.02, *p* < .05 with a medium effect size [*η*_*p*_^*2*^ = .13]), but not in the WLC group (*F*_*(1*, *42)*_ = .00, *p* = 1).

With respect to the psychophysiological reactions during the anger recall task, there was a significance interaction effect of Time*Group in RR (*F*_*(2*, *42)*_ = 3.34, *p* < .05 with a large effect size [*η*_*p*_^*2*^ = .14]) and a marginal effect in BVA (*F*_*(2*, *37)*_ = 3.21, *p* = .052 with a large effect size [*η*_*p*_^*2*^ = .15]). The simple main effect showed a significant decrease on RR in the FTF group (*F*_*(1*, *42)*_ = 6.90, *p* < .05 with a large effect size [*η*_*p*_^*2*^ = .14]) and Internet-based group (*F*_*(1*, *42)*_ = 14.12, *p* < .001 with a large effect size [*η*_*p*_^*2*^ = .25]), but not in the WLC group(*F*_*(1*, *42)*_ = .04, *p* = .843); it also showed a marginally increased BVA level in the FTF group (*F*_*(1*, *37)*_ = 3.05, *p* = .09 with a medium effect size [*η*_*p*_^*2*^ = .08]), and a significantly increased BVA level in the Internet-based group (*F*_*(1*, *37)*_ = 8.10, *p* < .01 with a large effect size [*η*_*p*_^*2*^ = .18]), but not in the WLC group(*F*_*(1*, *37)*_ = .83, *p* = .37).

## Discussion

Many patients with CAD in need cannot access FTF psychosocial rehabilitation due to excessive travel distance. Therefore, the iCBGT program was developed as an alternative through (1) adopting the synchronous OVC, which maintained the group interaction function similar to an FTF program, and (2) building asynchronous SLP to deliver psychoeducation content. Moreover, this pilot study used a small sample size of CAD patients (13, 18, and 14 for iCBGT, FTF, and WLC, respectively) to assess feasibility and acceptability, explore use patterns, and measure risk factor changes. No participants had depression. Results of the pilot study supported the feasibility and acceptability of the iCBGT using video conference for patients with CAD, with medium (anxiety, hostility, and vasodilation) to large (RR) effect sizes which were comparable with, if not better than, those of the traditional FTF program.

### Advantages of synchronous OVC sessions

By adopting an OVC system which contained videos of all the group members to enable immediate verbal and nonverbal responses (i.e., eye contact, smiling, forward posture), this program maintained the group interaction function of an FTF program but on the Internet. In addition, with the support of two orientation sessions, group members knew each other and had shared their traumatic experiences during disease onset in person. Thus, they acknowledged that they were communicating with real people on OVC, which shortened the psychological distance among group members despite their physical distance. Consequently, group leaders (clinical psychologists) were able to facilitate group interaction and reinforce cognitive reconstructions and self-compassionate thoughts and behaviors as they did in the traditional FTF group.

Group cohesion and a sense of universality was sustained by realizing that they have the same afflictions struggling with physical and occupational impairment as well as anxiety about death or recurrence. This support was vital for the morale of group members to encourage them to enhance psychological and cardiac health together, whereas individual Internet-based CBT [15] or mHealth-delivered self-management programs that only adopt an asynchronous website [14] are unable to offer this.

### Advantages of the Internet-based program combining both asynchronous and synchronous systems

Asynchronous SLP not only offered traditional FTF teaching materials online but also combined discussion through synchronous OVC sessions. This Internet-based program adopted the advantages of the “flipped classroom” [27]. With the support of the SLP, participants with CAD accessed the psychoeducation curriculum prior instead of waiting until they got into the session and received it directly from the therapists. Moreover, they could study the course content at their own pace according to their requirements, which encouraged participants to actively attain knowledge and thus potentially improving their learning efficiency [28] and treatment adherence.

Group leaders of the OVC sessions, with the support of SLP, had more time to clarify the problems participants encountered and ensure that they performed the techniques properly in group sessions compared with the traditional FTF program. Furthermore, when participants had questions about techniques and posted them on the discussion forum prior to every OVC session, group leaders had more time to prepare answers for each session. These timely responses from the leaders to the posted questions and difficulties encouraged participants and motivated them to visit the SLP more frequently. The median login no. (42, over 8 weeks) was higher than that (3, over 24 weeks) of the mHealth-delivered program that adopted automatically delivered text messages and an asynchronous website [14].

The average question no. was 1.77, which revealed that elderly participants were conservative about using the discussion forum. By adopting the synchronous OVC, group leaders interacted with all the elder participants such that all members could have more opportunity to give their experiences and questions about technique practices.

### Barriers and strength to participate in an iCBGT program for patients with CAD

Although many advantages exist for the Internet-based program, including a slightly lower dropout rate (23.5% vs. 30.8%), it is subject to some limitations. For example, CAD mostly occurs in elderly patients; however, computer and Internet usage is less prevalent in these patients than among younger generations. Computer and Internet literacy is a necessary condition to participate in the iCBGT. Consequently, in this study, based on their preference and familiarity with Internet use, fewer (39.5% vs. 60.5%) participants chose to participate in the iCBGT program. The ratio of male (92.3% vs. 50%), the ratio of employed (53.8% vs. 35.3%), and the total duration of education (13.9 vs. 12.5 years) in the iCBGT group were higher than those in the FTF group. These phenomena indicated that although iCBGT has time, space, and economic benefits, it also has accessibility problems. This finding is similar with the report of Steinmark, Dornelas and Fisher [29]. In addition to the gender difference in cardiological disease incidence, more men in this study chose to participate in iCBGT. The reason may be that employed men had limited time to participate in CBGT conducted FTF in hospitals or that the men with a longer education had information literacy required for SLP and OVC. Further research to validate these hypotheses is warranted. However, with the current prevalence of the Internet globally and expected growth, future older generations may have a higher acceptance for iCBGT.

### Treatment effectiveness of iCBGT programs

The anxiety (Mean = 43.52 ± 10.28, range from 21 to 66) and hostility (Mean = 53.78 ± 12.97, range from 21 to 83) levels of the participants with CAD at pre-intervention were measured as mild to moderate, whereas the depression levels (Mean = 7.47 ± 6.77, range from 0 to 28) were located within normal range. The results indicated that the iCBGT program was as efficacious as a traditional FTF program in reducing anxiety and hostility. However, both intervention programs showed no significant effect on depression. This may be due to the flooring effect caused by the normal depression level of the participants invited from cardiology clinics in this study. Glozier et al. [15] specially targeted adults with mild to moderate depression and high cardiovascular disease risks and did demonstrate an improvement in depressive symptoms through freely accessible Internet-based CBT. Whether iCBGT is applicable for patients with CAD with mild to moderate depression requires validation in a future study.

### Study limitations

The treatment effect of the Internet-based program developed in this study combined both asynchronous and synchronous systems; consequently, the effect of separate single system could not be clarified. For investigating the acceptance of iCBGT, a convenience sampling instead of RCT was adopted; furthermore, the sample size was small, thus limiting the generalizability of the study. Because this was a pilot study, the CAD severity, pharmacological treatment, and time since illness onset of the participants were not recorded. These factors could interfere with the measured parameters. Future studies should enroll more participants, adopt more rigorous RCT, and record the aforementioned parameters to improve research generalizability. This study only examined the immediate effect of iCBGT to reduce the psychological risk factors for patients with CAD. As such, a long-term follow-up study should be applied to examine whether the efficacy of iCBGT can be maintained long term and reduce the recurrence rate of cardiac events, re-hospitalization, and mortality among patients with CAD.

## Conclusion

This study is the first to develop an Internet-based CBGT program with both synchronous OVC and asynchronous SLP assistance for patients with CAD. The participants of the program were highly motivated and satisfied with visiting the self-learning platform and video conferences. Results indicated that Internet-based CBGT is feasible and acceptable for the psychosocial rehabilitation of patients with CAD, and provides an alternative for patients who are unable to obtain conventional FTF psychosocial rehabilitation.

## Supporting information

S1 Dataset Dataset for Table 2. (XLSX)
